# Expression of the chrXq27.3 miRNA cluster in recurrent ovarian clear cell carcinoma and its impact on cisplatin resistance

**DOI:** 10.1038/s41388-020-01595-3

**Published:** 2021-01-08

**Authors:** Kosuke Yoshida, Akira Yokoi, Mai Sugiyama, Shingo Oda, Kazuhisa Kitami, Satoshi Tamauchi, Yoshiki Ikeda, Nobuhisa Yoshikawa, Kimihiro Nishino, Kaoru Niimi, Shiro Suzuki, Fumitaka Kikkawa, Tsuyoshi Yokoi, Hiroaki Kajiyama

**Affiliations:** 1 Department of Obstetrics and Gynecology, Nagoya University Graduate School of Medicine, Nagoya, Japan; 2 Institute for Advanced Research, Nagoya University, Nagoya, Japan; 3 Department of Drug Safety Sciences, Division of Clinical Pharmacology, Nagoya University Graduate School of Medicine, Nagoya, Japan; 4 Bell Research Center, Department of Obstetrics and Gynecology Collaborative Research, Nagoya University Graduate School of Medicine, Nagoya, Japan

**Keywords:** Ovarian cancer, Epigenomics

## Abstract

Ovarian clear cell carcinoma (OCCC) is a histological subtype of epithelial ovarian cancer and exhibits dismal prognosis due to chemoresistance. Moreover, only few effective therapeutic options exist for patients with recurrent OCCC, and an understanding of its molecular characteristics is essential for the development of novel therapeutic approaches. In the present study, we investigated unique MicroRNAs (miRNA) profiles in recurrent/metastatic OCCC and the role of miRNAs in cisplatin resistance. Comprehensive miRNA sequencing revealed that expression of several miRNAs, including miR-508-3p, miR-509-3p, miR-509-3-5p, and miR-514a-3p was remarkably less in recurrent cancer tissues when compared with that in paired primary cancer tissues. These miRNAs are located in the chrXq27.3 region on the genome. Moreover, its expression was negative in omental metastases in two patients with advanced OCCC. In vitro analyses revealed that overexpression of miR-509-3p and miR-509-3-5p reversed cisplatin resistance and yes-associated protein 1 (YAP1) was partially responsible for the resistance. Immunohistochemistry revealed that YAP1 expression was inversely correlated with the chrXq27.3 miRNA cluster expression. In conclusion, these findings suggest that alteration of the chrXq27.3 miRNA cluster could play a critical role in chemoresistance and miRNAs in the cluster and their target genes can be potential therapeutic targets.

## Introduction

Epithelial ovarian cancer (EOC) remains one of the leading causes of cancer death among females worldwide, being responsible for an estimated 151,900 deaths in 2012 [1]. High-grade serous carcinoma (HGSOC) is the most common type of EOC, and ovarian clear cell carcinoma (OCCC) is more commonly diagnosed in Asian countries [2, 3]. OCCC is known to exhibit greater chemoresistance than HGSOC [2, 3]. In general, patients with EOC undergo cytoreductive surgery combined with platinum-containing chemotherapy, but some patients eventually develop platinum-resistant disease [3]. Few effective therapeutic options exist for patients with platinum-resistant EOC [3]. Therefore, it is important to know the molecular characteristics of recurrent EOC, and novel therapeutic approaches for recurrent EOC are highly demanded. However, these efforts are hindered by the difficulty in obtaining recurrent cancer tissues because surgery is not a standard approach for patients with recurrent EOC.

MicroRNAs (miRNAs), small noncoding RNA molecules consisting of ~22 nucleotides, regulate gene expression posttranscriptionally and play multiple roles in various processes including cancer progression and drug resistance [4–10]. MiRNA clusters contain a set of two or more miRNA-encoding genes, and 159 miRNA clusters have been reported in the human genome [11]. In addition, many miRNA genes are located inside or close to fragile sites, including one miRNA cluster located in the chrXq27.3 region [5, 8, 12]. The region is the key spot for fragile X syndrome, the most common form of hereditary intellectual disability, but the association between the miRNA cluster and fragile X syndrome remains unknown [12, 13]. In addition, all 22 miRNA genes in the chrXq27.3 cluster are oriented in the same transcriptional direction, and there are no annotated protein-coding genes interrupting the miRNA genes in the cluster [12]. Hence, these miRNAs are considered to be under the control of a common regulatory unit and co-expressed [7, 14]. Recently, several reports described the roles of miRNAs belonging to the cluster in various cancers [15–19]. In EOC, several reports indicated an association between the status of the miRNA cluster and clinical outcomes [20–22]. However, these reports mostly included patients with HGSOC, whereas few patients with OCCC were enrolled. Especially, tissue samples of recurrent OCCC are rare, and therefore, the function of the cluster in recurrent OCCC remains unknown.

In the present study, we identified remarkably decreased expression of chrXq27.3 miRNA cluster in recurrent and metastatic OCCC. Subsequently, in vitro analyses demonstrated that miR-509-3p and miR-509-3-5p, two members of the chrXq27.3 cluster, are associated with cisplatin resistance via yes-associated protein 1 (YAP1) and the Hippo signaling pathway.

## Results

### Identification of the miRNA profiles of recurrent OCCC

First, comprehensive miRNA sequencing was performed using formalin-fixed, paraffin-embedded (FFPE) tissues. We included 20 patients with stage I OCCC, and a summary of the patients’ characteristics is shown in Table 1. Cases 1–10 experienced recurrence, and cases 1, 3–5, and 8 underwent secondary surgery. The progression-free survival (PFS) times of the five aforementioned patients were 19.6, 47.9, 17.9, 9.9, and 17.8 months, respectively, and local recurrence was only observed in case 5. Conversely, cases 11–20 have remained cancer-free since the initial treatment.

**Table 1:** Patients’ characteristics.

No.	Sample	Age	Stage	Initial surgery	Chemotherapy	Recurrence site	Secondary surgery	PFS (months)
1	FFPE	39	IA	ATH + BSO + OM + PEN + PAN	TC	Pelvic lymph nodes	Yes	19.6
2	FFPE	57	IC3	ATH + BSO + OM + PEN + PAN	TC	Multiple lymph nodes and brain	No	37.6
3	FFPE	64	IC3	ATH + BSO + OM + PEN + PAN	TC	Peritoneal dissemination	Yes	47.9
4	FFPE	51	IA	ATH + BSO + OM + PEN + PAN	TC	Lung	Yes	17.9
5	FFPE	38	IC1	ATH + BSO + OM + PEN + PAN	TC	Vaginal stump	Yes	9.9
6	FFPE	65	IC1	ATH + BSO + OM + PEN + PAN	TC	Peritoneal dissemination	No	2.1
7	FFPE	48	IC1	ATH + BSO + OM + PEN + PAN	TC	Peritoneal dissemination	No	41.3
8	FFPE	70	IC1	ATH + BSO + OM	TC	Para-aortic lymph nodes	Yes	17.8
9	FFPE	41	IC1	ATH + BSO + OM	TC	Para-aortic lymph nodes	No	22.6
10	FFPE	59	IC3	ATH + BSO + OM	TC	Multiple lymph nodes	No	11.4
11	FFPE	36	IC1	ATH + BSO + OM + PEN + PAN	TC	No recurrence	–	83.7
12	FFPE	57	IC3	ATH + BSO + OM + PEN + PAN	TC	No recurrence	–	66.1
13	FFPE	64	IC3	ATH + BSO + OM + PEN + PAN	TC	No recurrence	–	67.0
14	FFPE	53	IC1	ATH + BSO + OM + PEN + PAN	TC	No recurrence	–	127.4
15	FFPE	44	IC1	ATH + BSO + OM + PEN + PAN	TC	No recurrence	–	61.6
16	FFPE	64	IC1	ATH + BSO + OM + PEN + PAN	TC	No recurrence	–	35.6
17	FFPE	49	IC3	ATH + BSO + OM + PEN + PAN	TC	No recurrence	–	54.3
18	FFPE	68	IC3	ATH + BSO	TC	No recurrence	–	29.0
19	FFPE	39	IA	ATH + BSO + OM	TC	No recurrence	–	65.3
20	FFPE	62	IC1	ATH + BSO + OM + PEN + PAN	DC	No recurrence	–	82.4
21	Fresh/PDX	49	IC3	ATH + BSO	TC	No recurrence	–	25.5
22	Fresh/PDX	62	IC1	ATH + BSO + OM + PEN + PAN	–	No recurrence	–	23.3
23	Fresh/PDX	37	IA	LSO + Rt. Cystectomy + OM + PEN biopsy	TC	No recurrence	–	24.8
24	Fresh	49	IIIC	ATH + BSO + OM	TCB	Portal vein lymph nodes	No	3.7
25	Fresh	67	IIIC	Partial BSO + Partial OM	–	Residual lesion	No	0.0

*FFPE* formalin-fixed paraffin-embedded tissues, *PDX* patient-derived xenograft, *ATH* abdominal total hysterectomy, *BSO* bilateral salpingo-oophorectomy, *OM* omentectomy, *PEN* pelvic lymph node dissection, *PAN* para-aortic lymph node dissection, *TC* paclitaxel and carboplatin, *DC* docetaxel and carboplatin, *TCB* TC and bevacizumab, *PFS* progression-free survival.

**Fig. 1:**
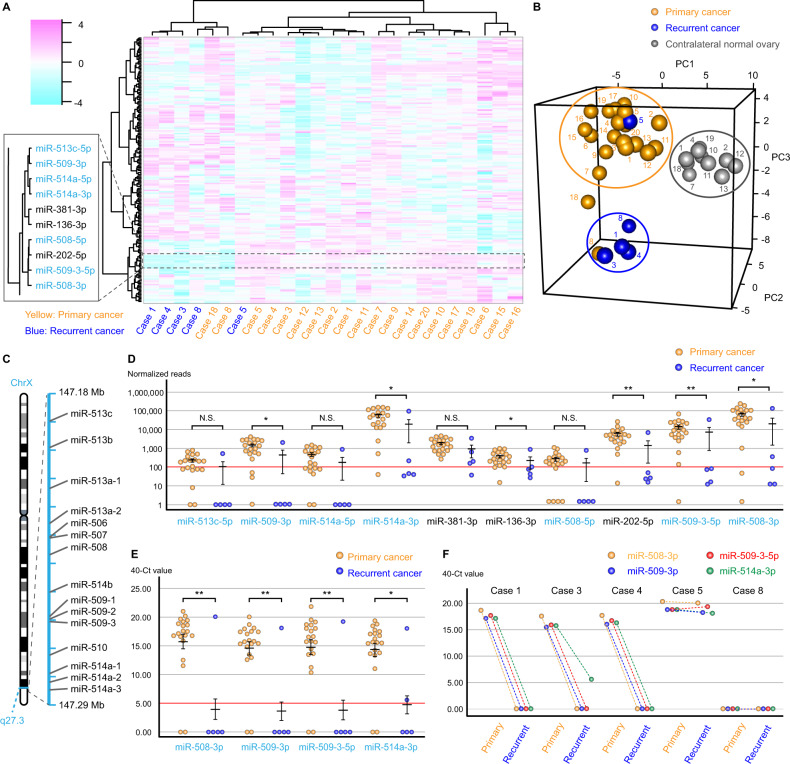
The miRNA profiles of recurrent and primary OCCC samples. **A** The hierarchical clustering and heatmap and **B** principal component analysis of the miRNA profiles of FFPE tissues from 20 patients with primary cancer (cases 1–20), including five recurrent cancers (cases 1, 3–5, and 8) and ten contralateral normal ovarian tissues (cases 1, 2, 4, 7, 10–13, 18, and 19). **C** The location of miRNA precursors in the chrXq27.3 cluster according to the online database, RNAcentral. **D** The normalized reads of the downregulated miRNAs in recurrent carcinomas. The primary and recurrent carcinomas were compared using the chi-squared test at a threshold of 100 reads. **E** qPCR analysis of four miRNAs in primary and recurrent carcinomas. The primary and recurrent carcinomas were compared using the chi-squared test at a threshold of 5.00 “40—Ct value.” **F** Comparison of the expression of four miRNAs was measured using qPCR in paired primary and recurrent carcinoma samples. Error bars represent the mean with standard errors of the mean. N.S. not significant; *p < 0.05 and **p < 0.01.

Heatmap and principal component analysis revealed that miRNA profiles of recurrent cancer in cases 1, 3, and 4 were similar profile (Fig. 1A, B). Moreover, similar miRNA profiles were also found for the primary and recurrent cancers of case 8 and the primary cancer of case 18. However, the recurrent cancer profile of case 5 was similar to that of the paired primary cancer. Then, comparing paired primary and recurrent cancers from case 1, 3, and 4, we identified commonly dysregulated miRNAs, and according to the heatmap analysis, ten miRNAs downregulated in patients with recurrent cancer were clustered in the same group (Fig. 1A). Moreover, seven of the miRNAs are located in the chrXq27.3 region (Fig. 1C). Furthermore, miR-509-3p, miR-514a-3p, miR-136-3p, miR-202-5p, miR-509-3-5p, and miR-508-3p were significantly downregulated in recurrent cancers compared with their expression in primary cancers (*p < 0.05 and **p < 0.01; Fig. 1D). Thus, we focused on four miRNAs in the cluster: miR-508-3p, miR-509-3p, miR-509-3-5p, and miR-514a-3p. Then, the results were validated using quantitative polymerase chain reaction (qPCR), and all four miRNAs were significantly downregulated in recurrent cancers compared with their expression in primary cancers (*p < 0.05 and **p < 0.01; Fig. 1E). Repeatedly, all recurrent cancers, excluding that in case 5, exhibited no expression of those four miRNAs (Fig. 1F).

### Expression of the chrXq27.3 miRNA cluster in advanced OCCC and patient-derived xenograft (PDX) models

**Fig. 2:**
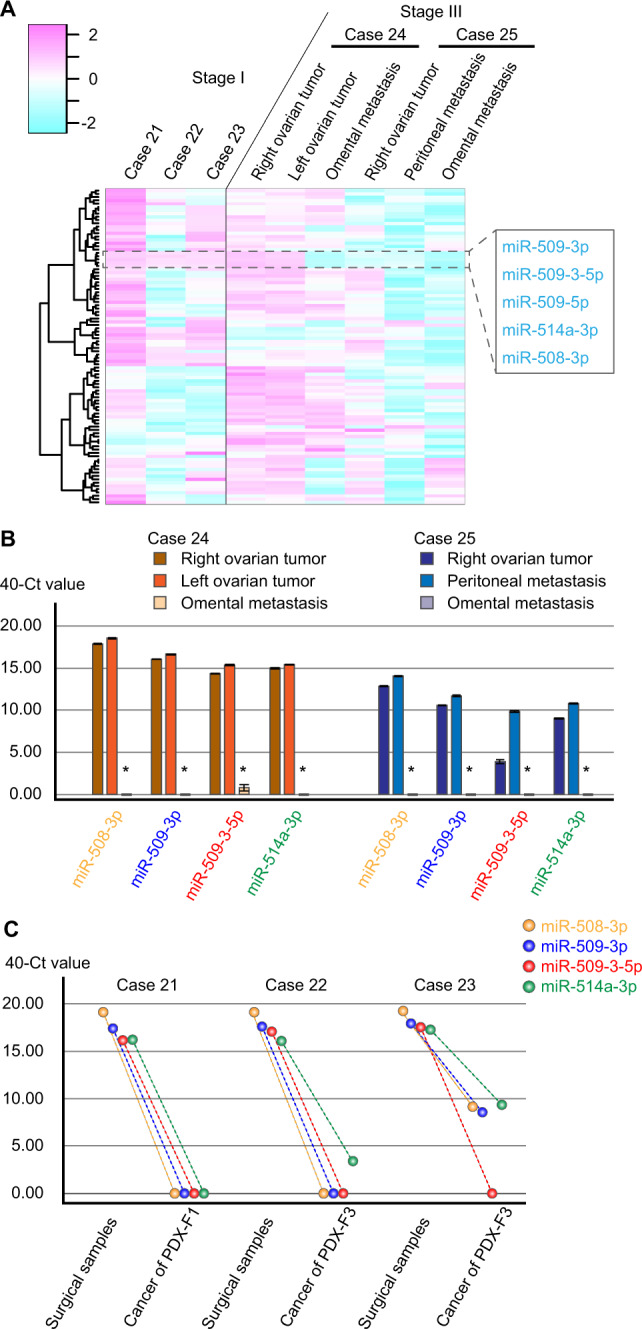
The status of the chrXq27.3 miRNA cluster in metastatic OCCC and PDX models. **A** The hierarchical clustering and heatmap analysis in fresh-frozen tissues of OCCC. **B** The expression of four miRNAs in primary and metastatic cancers was measured using qPCR and compared with Kruskal–Wallis test. Error bars represent the mean with standard errors of the mean, *p < 0.05. **C** Comparison of the expression of four miRNAs in surgical samples and PDX models as measured by qPCR.

To validate the results of FFPE samples, we analyzed the miRNA profiles of five fresh-frozen OCCC tissues. As shown in Table 1, cases 21–23 had stage I OCCC, and cases 24 and 25 had stage III OCCC. Case 24 received neoadjuvant chemotherapy followed by interval debulking surgery, and bilateral ovarian and omental metastases were resected. Case 25 underwent exploratory laparotomy and ovarian, peritoneal, and omental metastases were resected. The heatmap analysis revealed that the miRNAs in the chrXq27.3 cluster were clustered into the same group and downregulated in metastatic OCCC (Fig. 2A). This result was also validated by qPCR analysis, which revealed that the four miRNAs in the cluster were significantly downregulated in omental metastases compared with their expression in other tissues in cases 24 and 25 (*p* < 0.05 in all miRNAs in both cases, Fig. 2B).

Then, we established three PDX models from cases 21–23, and qPCR analysis demonstrated that after implanting tumors in mice, the expression of the four miRNAs decreased dramatically (Fig. 2C).

### The function of chrXq27.3 miRNA cluster in cisplatin resistance

**Fig. 3:**
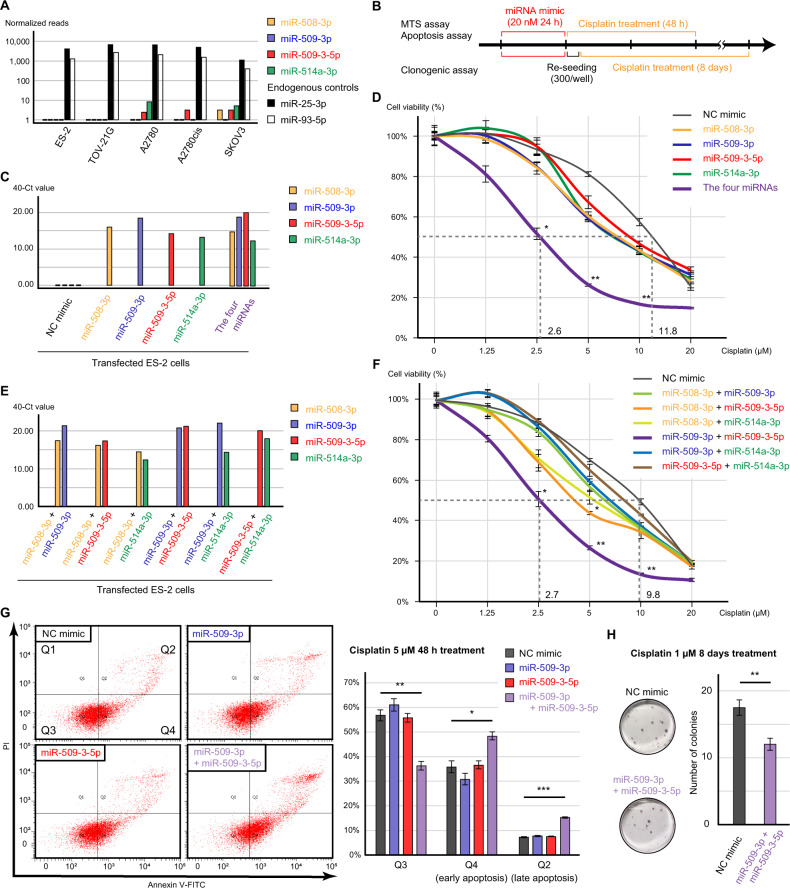
The status of the chrXq27.3 miRNA cluster in a preclinical model and its function in cisplatin sensitivity. **A** Expression of the chrXq27.3 miRNA cluster in several cell lines. miR-25-3p and miR-93-5p are presented as endogenous controls. **B** The schema of cisplatin-resistant analysis. ES-2 cells were transfected with 20 nM miRNA mimic for 24 h, and subsequently cells were treated with cisplatin containing medium (0, 1.25, 2.5, 5, 10, and 20 μM) for 48 h. **C** Expression of the chrXq27.3 miRNA cluster in transfected ES-2 cells measured using qPCR. **D** Cisplatin sensitivity of transfected ES-2 cells measured using the MTS assay. The viability of cells following transfection of each miRNA was compared with that of negative control (NC) mimic-transfected cells using the Kruskal–Wallis test at each cisplatin concentration. **E** Expression of the chrXq27.3 miRNA cluster in co-transfected ES-2 cells measured using qPCR. **F** Cisplatin sensitivity of co-transfected ES-2 cells measured using the MTS assay. The viability of cells following transfection of each miRNA was compared with that of NC mimic-transfected cells using the Kruskal–Wallis test at each cisplatin concentration. **G** Apoptosis assay of transfected cells treated with 5 µM cisplatin. The percentage of apoptotic cells was compared using Student’s *t* test. **H** Representative images and bar graph of clonogenic assay. The transfected cells were treated with 1 µM cisplatin for 8 days, and the number of colonies was compare using Student’s *t* test. Error bars represent standard errors of the mean. *p < 0.05, **p < 0.01, and ***p < 0.001.

The expression of the chrXq27.3 miRNA cluster was downregulated in several EOC cell lines, whereas the expression of the endogenous miRNAs miR-25-3p and miR-93-5p was retained [23] (Fig. 3A). Thus, we evaluated the cisplatin sensitivity using miRNA-transfected ES-2 cells because patients with OCCC exhibit chemoresistance (Fig. 3B). miRNA mimics for miR-508-3p, miR-509-3p, miR-509-3-5p, and miR-514a-3p were successfully transfected, and the negative control (NC) mimic had no effect on the expression of the miRNAs (Fig. 3C). However, there were no obvious differences in cisplatin sensitivity among the miRNA-transfected cells (Fig. 3D). Based on the data from clinical samples of primary tumors, we transfected all four miRNAs concurrently, and this co-transfection resulted in significantly decreased cell viability in the presence of 2.5, 5, and 10 μM cisplatin (*p* < 0.05, *p* < 0.01, and *p* < 0.01, respectively, Fig. 3C, D). The 50% inhibitory concentration (IC50) of cisplatin in cells transfected with four miRNAs was 24.1% of that in cells transfected with the NC mimic. To further evaluate this reversal of cisplatin resistance, we transfected cells with different combinations of the miRNAs, and co-transfection of miR-509-3p and miR-509-3-5p was linked to significantly decreased cell viability in the presence of 2.5, 5, and 10 μM cisplatin (*p* < 0.05, *p* < 0.01, and *p* < 0.01, respectively, Fig. 3E, F). Similarly, the IC50 in cells co-transfected with miR-509-3p and miR-509-3-5p decreased to 27.6% of the value in cells transfected with the NC mimic. This result was also confirmed through an apoptosis assay, and the rate of cisplatin-induced apoptosis was significantly increased by the co-transfection of miR-509-3p and miR-509-3-5p (early and late apoptosis, *p* < 0.05 and *p* < 0.001, respectively, Fig. 3G). Additionally, clonogenic assay showed that the combination of the miRNAs significantly suppressed cell survival during long-term cisplatin exposure (Fig. 3H).

To validate the impact of the two miRNAs, we performed experiments using A2780cis cell line. As expected, co-transfection of miR-509-3p and miR-509-3-5p significantly decreased cell viability in the presence of 5, 10, 20, and 40 μM cisplatin (*p* < 0.05, *p* < 0.01, *p* < 0.05, and *p* < 0.01, respectively, Supplementary Fig. 1A, B). Moreover, the co-transfection significantly increased the rate of apoptotic cells (early and late apoptosis, *p* < 0.01 and *p* < 0.05, respectively, Supplementary Fig. 1C).

### Identification of the target genes of miR-509-3p and miR-509-3-5p

**Fig. 4:**
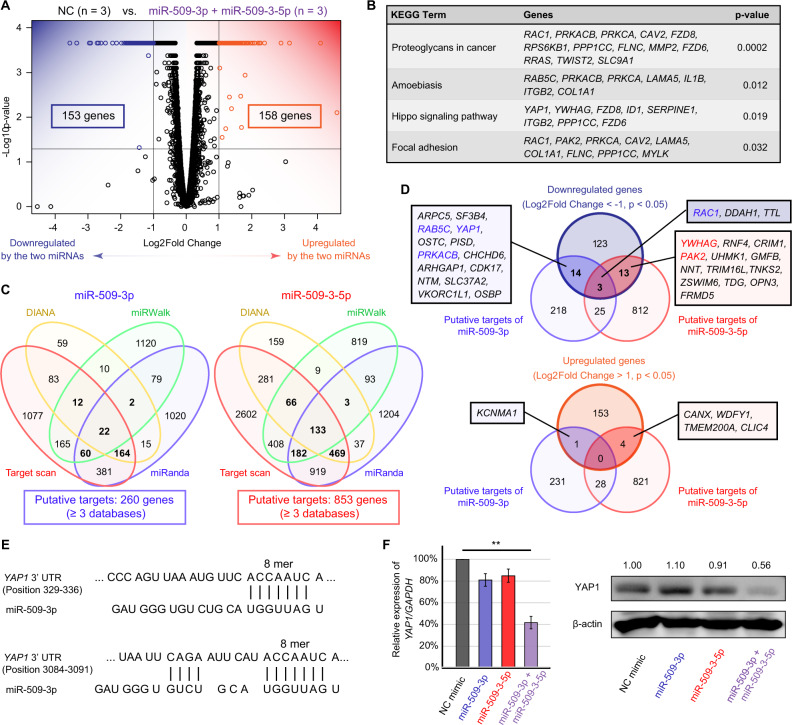
Dysregulated genes following co-transfection of miR-509-3p and miR-509-3-5p and target prediction. **A** Volcano plot of dysregulated genes generated via mRNA sequencing of transfected cells. Genes expressed at <10 FPKM both in both negative control-transfected and co-transfected ES-2 cells were excluded. **B** Functional annotation of the dysregulated genes by following co-transfection in terms of KEGG pathways. **C** Number of putative targets of miR-509-3p or miR-509-3-5p. The online programs TargetScanHuman7.2, DIANA-microT-CDS, miRWalk3.0, and miRanda were used. **D** Venn diagrams for dysregulated genes and putative targets of the miRNAs. **E** Predicted interaction between YAP1 and miR-509-3p. **F** Validation of YAP1 suppression following co-transfection of miR-509-3p and miR-509-3-5p at the transcriptional and protein levels. Relative expression of *YAP1/GAPDH* was compared using Student’s *t* test (**p < 0.01).

To identify the target genes of miR-509-3p and miR-509-3-5p, we performed mRNA sequencing using ES-2 cells. Compared with transfection of the NC mimic, co-transfection of miR-509-3p and miR-509-3-5p significantly decreased and increased the expression of 153 and 158 genes, respectively (Fig. 4A). Functional annotation of the dysregulated genes revealed four significantly dysregulated Kyoto Encyclopedia of Genes and Genomes (KEGG) pathways, including Hippo signaling pathway (*p* = 0.019, Fig. 4B). Then, the putative targets of miR-509-3p and miR-509-3-5p were explored using four algorithms: TargetScanHuman7.2, DIANA-microT-CDS, miRWalk3.0, and miRanda. The genes selected in at least three databases were considered putative targets. In total, 260 genes were targeted by miR-509-3p, and 853 genes were targeted by miR-509-3-5p (Fig. 4C). In addition, genes identified using both mRNA sequencing and target predictions were evaluated, including 30 downregulated and 5 upregulated genes (Fig. 4D). Of these genes, we focused on YAP1, a gene involved in the Hippo signaling pathway that was highly expressed in NC mimic-transfected cells (Fig. 4B, D and Supplementary Table S1). TargetScanHuman7.2 revealed that YAP1 has two binding sites for miR-509-3p in its 3′-untranslated regions (Fig. 4E). YAP1 is not regard as the putative target of miR-509-3-5p even though miRWalk3.0 identified a binding site for miR-509-3-5p in its cording region (1371–1411). In addition, miR-509-3-5p contributed to the dysregulation of the Hippo signaling pathway by targeting tyrosine 3-monooxygenase/tryptophan 5-monooxygenase activation protein gamma (YWHAG, also known as 14-3-3gamma; Fig. 4B, D)

Then, the result of gene expression was validated by qPCR and Western blotting. Co-transfection of miR-509-3p and miR-509-3-5p decreased YAP1 expression at both transcript and protein levels, whereas transfection of miR-509-3p or miR-509-3-5p alone did not alter YAP1 expression (Fig. 4F and Supplementary Fig. 1D).

### The impact of YAP1 on cisplatin sensitivity

**Fig. 5:**
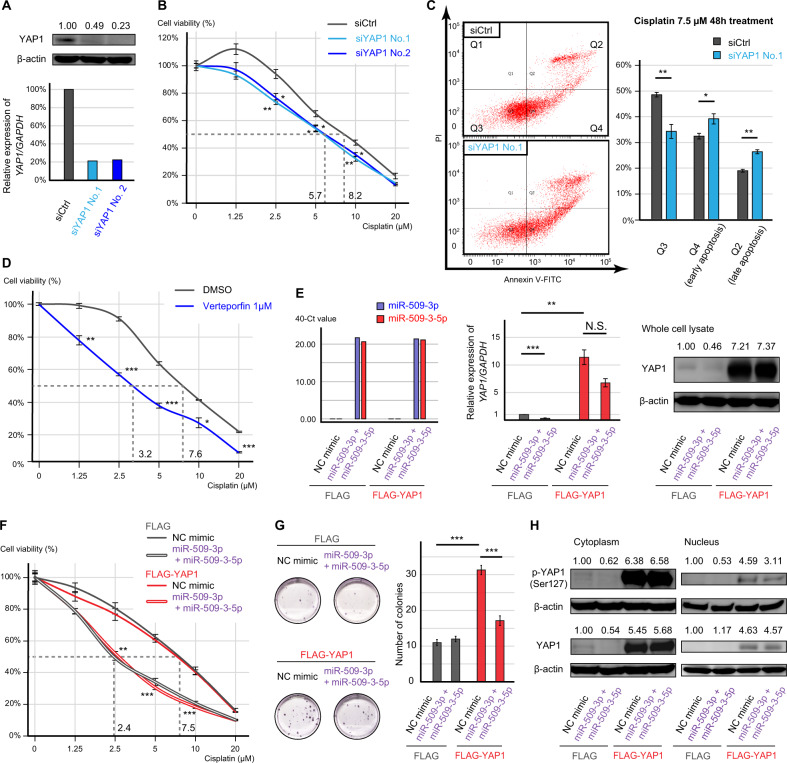
The effect of YAP1 on cisplatin resistance in vitro. **A** Transfection efficacy of two siRNAs for YAP1 (siYAP1 No.1 and No.2). **B** Cisplatin sensitivity of siYAP1-transfected ES-2 cells measured using MTS assay. The viability of treated cells was compared with that of cells transfected with negative control siRNA (siCtrl) using Student’s *t* test. **C** Apoptosis assay of transfected cells treated with 7.5 µM cisplatin. The percentage of apoptotic cells was compared using Student’s *t* test. **D** Cisplatin sensitivity of YAP1 inhibitor-treated ES-2 cells measured using MTS assay. The viability of treated cells was compared with that of cells treated with DMSO using Student’s *t* test. **E** Expression of miRNAs and YAP1 in YAP1 overexpressed ES-2 cells (FLAG-YAP1) and mock transfected cells (FLAG). The cells were further transfected with combination of miR-509-3p and miR-509-3-5p. **F** Cisplatin sensitivity of FLAG-YAP1 cells measured using MTS assay. The viability of the two miRNAs transfected cells was compared with that of NC transfected cells using Student’s *t* test. **G** Representative images and bar graph of clonogenic assay. The transfected cells were treated with 1 µM cisplatin for 8 days, and the number of colonies was compare using Student’s *t* test. **H** The expression of phospho-YAP (Ser127) in the cytoplasm and nucleus of FLAG-YAP1 cells. Error bars represent standard errors of the mean. *p < 0.05, **p < 0.01, and ***p < 0.001.

To evaluate the effect of YAP1 on cisplatin sensitivity in OCCC, we performed a gene silencing assay using small-interfering RNAs (siRNAs) or an inhibitor targeting YAP1. Two siRNAs for YAP1 (siYAP1) decreased the expression of YAP1 and significantly decreased cell viability in the presence of 2.5, 5, and 10 μM cisplatin (siCtrl vs. siYAP1 No.1, *p* < 0.01, *p* < 0.05, and *p* < 0.01, respectively, and siCtrl vs. siYAP1 No.2, *p* < 0.05 for all concentrations, Fig. 5A, B and Supplementary Fig. 1E). Furthermore, the rates of early and late apoptotic cells were significantly increased by siYAP1 No.1 in ES-2 cells (*p* < 0.05 and *p* < 0.01, respectively, Fig. 5C). Moreover, 1 μM verteporfin, a YAP1 inhibitor, significantly decreased cell viability irrespective of the cisplatin concentration (1.25, 2.5, 5, 10, and 20 μM cisplatin, *p* < 0.01, *p* < 0.001, *p* < 0.001, *p* < 0.05, and *p* < 0.001, respectively, Fig. 5D).

Then, we established stable YAP1 overexpressed ES-2 cells (FLAG-YAP1). When compared with mock transfected cells (FLAG), the FLAG-YAP1 cells highly expressed YAP1 even after transfecting the two miRNAs (Fig. 5E). However, in FLAG-YAP1 cells, co-transfection of miR-509-3p and miR-509-3-5p significantly decreased cell viability in the presence of 2.5, 5, and 10 μM cisplatin (*p* < 0.01, *p* < 0.001, and *p* < 0.001, respectively, Fig. 5F). In addition, clonogenic assay showed that the two miRNAs significantly decreased the number of colonies in FLAG-YAP1 cells (*p* < 0.001), although FLAG-YAP1 cells showed significantly high colony forming ability than FLAG cells (*p* < 0.001, Fig. 5G). Therefore, YAP1 overexpression could not completely rescue the effect of the miRNAs. To identify the detailed status of YAP1 protein in the cells, additional Western blotting analysis was performed. The expression of YAP1 protein was remarkably high in both cytoplasm and nucleus in the FLAG-YAP1 cells although Ser127-phosphorylated YAP1 (p-YAP1) also demonstrated a remarkable increase (Fig. 5H).

### Clinical relevance of YAP1 in OCCC

**Fig. 6:**
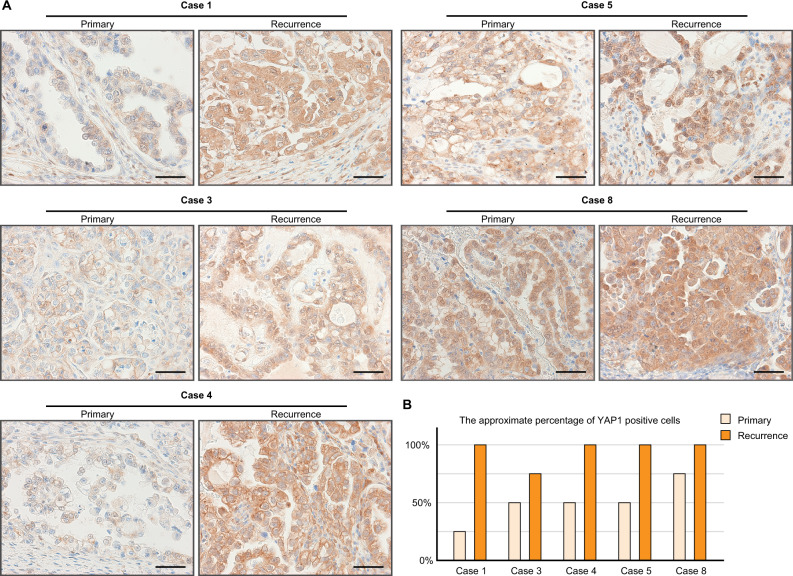
The expression of YAP1 in clinical samples. **A** Representative images of immunohistochemistry of YAP1. Scale bars show 50 μm. **B** The percentage of YAP1-positive cells.

Finally, to confirm the interaction between the chrXq27.3 miRNA cluster and YAP1, we performed immunohistochemistry of YAP1 using samples from cases 1, 3–5, and 8. As described previously, the expression of the cluster became negative in recurrent cancer in cases 1, 3, and 4. In these cases, YAP1 expression was obviously stronger in recurrent cancer than in primary cancer (Figs. 1F and 6A). Moreover, YAP1 expression in case 8 was strong in both primary and recurrent lesion, which was consistent with the decreased expression of the cluster (Figs. 1F and 6A). However, YAP1 expression in case 5 was also strong despite the positive expression of the cluster (Figs. 1F and 6A). In addition, in all cases, the percentage of YAP1-positive cells tended to be higher in recurrent cancer than in primary cancer (Fig. 6B).

## Discussion

**Fig. 7:**
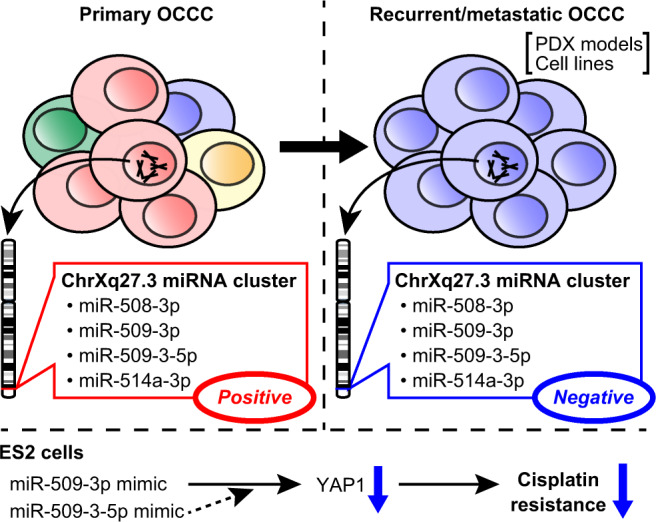
Graphical abstract. The expression of miRNAs in the chrXq27.3 miRNA cluster, which were abundant in primary ovarian clear cell carcinoma (OCCC), decreased in recurrent/metastatic OCCC. Moreover, their expression was also negative in several patient-derived xenograft (PDX) models and cell lines. In ES-2 cells, miR-509-3p and miR-509-3-5p reversed cisplatin resistance by coordinately regulating the expression of YAP1.

There are 22 miRNAs located in the chrXq27.3 miRNA cluster, but its detailed functions in EOC remain unclear [9, 10, 12]. In this study, we revealed the status of the chrXq27.3 miRNA cluster in OCCC and its function in cisplatin sensitivity (Fig. 7)

First, we uncovered that expression of the chrXq27.3 miRNA cluster was almost negative in recurrent and omental metastatic OCCC. Exceptionally, only one recurrent cancer (case 5) retained expression of this cluster. Given clinical information such as short PFS and the intraoperative findings of case 5, we considered the recurrent cancer to be a residual lesion of the primary cancer, and hence, they featured extremely similar miRNA profiles. In IHC, despite the high expression of the miRNAs, YAP1 expression was not suppressed in case 5. Therefore, the chrXq27.3 cluster independent regulation of YAP1 may be observed in about 20% of OCCC. Moreover, increased YAP1 expression may be suitable for survival because the percentage of YAP1-positive cells increased after recurrence in all cases.

Previous reports demonstrated that low expression of miRNAs in the chrXq27.3 cluster was associated with poor survival, and miRNA expression in the cluster was lower in recurrent cancer than in primary HGSOC [20–22]. However, reflecting histological differences, the expression of the cluster was overexpressed in primary OCCC when compared with HGSOC [24]. Therefore, the miRNAs may be associated with chemoresistance of recurrent cancer rather than OCCC-specific chemoresistance.

One of the interesting findings of this study is the profile of the chXq27.3 miRNA cluster in preclinical models. The advantage of the PDX model is that its characteristics are similar to those of the original tumor [25, 26]. However, we revealed the absence of chrXq27.3 miRNA cluster expression in PDX models, and thus, the PDX models may not be able to recapitulate the expression of these miRNAs. We assumed that cancer cells lacking this cluster have high tolerance to stress, and they could survive in mice. However, it is difficult to evaluate whether the PDX tumors acquired chemoresistance because there are no suitable control tumors expressing these miRNAs. In addition, consistent with previous reports, the expression of the miRNAs in the cluster was also negative in several EOC cell lines [22, 27]. Thus, downregulation of the cluster might be suitable for surviving in stressful culture dishes. Reportedly, miR-508-3p expression is negatively correlated with stemness and epithelial-mesenchymal transition-associated gene expression [18, 28]. However, the transcriptional regulation of the cluster under stressful conditions has not been elucidated. Therefore, the mechanisms of downregulation of the cluster in these preclinical models need further evaluation.

To evaluate the function of the miRNAs, single-stranded miRNA mimics were used in this study. Similar to the effects of typical double-stranded miRNA mimics, single-stranded miRNA mimics can also silence the expression of target genes [29, 30]. One advantage of single-stranded miRNA mimics is the reduction of potential off-target effects caused by the passenger strand of duplex RNA [30, 31]. The direct interaction between miR-509-3p and YAP1 was experimentally validated [22, 27]. However, we revealed that miR-509-3p and miR-509-3-5p coordinately regulated YAP1 expression in ES-2 cells. Considering a feature of miRNA clusters that members of the cluster have the same targets or target different genes belonging to specific pathways, this phenomenon suggested a new coordinate function of this cluster [11, 14]. About the role of miR-509-3-5p, there are several hypotheses. First, miR-509-3-5p can also directory regulate YAP1 expression by targeting its coding region because the miRNA targets situated in the coding region can be functional [6, 7]. Therefore, the expression of YAP1 may be more strongly suppressed by being targeted by two miRNAs. Second, the target genes of miR-509-3-5p may be important for regulation of YAP1. Otherwise, YAP1 expression was regulated by an unknown mechanism of the miRNAs. Overall, the coordinated regulation of YAP1 by the two miRNAs was a novel finding.

Platinum analogs are widely used and effective treatment for cancer [32]. However, cancer eventually acquires platinum resistance through multiple mechanisms [9, 26, 32]. According to previous reports, miR-509-3p expression is downregulated in cisplatin-resistant EOC, and miR-509-3p enhances cisplatin sensitivity in several EOC cell lines through various targets [33–35]. Conversely, the association between miR-509-3-5p and cisplatin resistance is incompletely understood, but miR-509-3-5p expression sensitized lung cancer cells to cisplatin in vitro [19]. Therefore, both miR-509-3p and miR-509-3-5p functions tumor suppressor.

Our study indicated that YAP1 is a key regulator of cisplatin sensitivity in OCCC. The Hippo signaling pathway regulates the activities of YAP1, which is involved in numerous cell-autonomous functions such as proliferation, stem cell properties, metabolism, and metastasis [36–38]. In addition, YAP1 promotes resistance to chemotherapy, targeted therapy, and hormone therapy through the upregulation of several pro-survival and antiapoptotic genes, and they also influence responses to immunotherapy by modulating the tumor immune microenvironment [37, 38]. In lung and ovarian cancer cells, YAP1 downregulation enhanced cisplatin sensitivity [36, 39]. Moreover, YAP1 expression was higher in metastasized tumors than in primary tumors in patients with EOC, and its higher expression was correlated with poor prognosis [40]. Therefore, downregulation of YAP1 enhanced cisplatin sensitivity. However, YAP1 overexpression could not rescue the effects of miR-509-3p and miR-509-3-5p, and one of the reasons for this may be the increased expression of p-YAP1 protein in FLAG-YAP1 cells. Reportedly, p-YAP protein is recognized by 14-3-3 protein and is sequestered within the cytoplasm, and therefore, our model may not fully enhance YAP1 [38]. Otherwise, modulation of the Hippo signaling pathway by other target genes may be enough to enhance cisplatin sensitivity.

There were several limitations in this study. First, we only evaluated a few patients with recurrent/metastatic OCCC. This is because patients with recurrent EOC rarely undergo surgery, and therefore, the status of the cluster should be evaluated in large-scale study. Second, the mechanism underling the decreased expression of the cluster in recurrent cancer remains unknown. A previous report showed that miR-506-3p, which is also a member of the cluster, was downregulated due to hypermethylation of its promoter region in pancreatic cancer [41]. Therefore, the expression of other miRNAs in the cluster may be regulated by the same mechanism. Moreover, we hypothesized that cancer cells in the primary site lacking the cluster could metastasize, survive, and eventually relapse. Single-cell sequencing may contribute to the elucidation of the mechanism. Third, the functions of the chrXq27.3 miRNA cluster are too numerous and complex to evaluate in a single study. Thus, we focused only on the roles of miR-509-3p and miR-509-3-5p in cisplatin resistance. Other functions of the cluster are worth evaluating, and we have planned studies in this aim.

In conclusion, we revealed that miR-508-3p, miR-509-3p, miR-509-3-5p, and miR-514a-3p were remarkably downregulated in recurrent and metastatic OCCC. In vitro analyses revealed that co-transfection of miR-509-3p and miR-509-3-5p enhanced cisplatin-induced apoptosis and induced the dysregulation of the Hippo signaling pathway, including YAP1 downregulation. This study revealed the involvement of the chrXq27.3 miRNA cluster in cisplatin sensitivity. We believe that the chrXq27.3 miRNA cluster has further important roles in cancer progression, making it a potential therapeutic target in OCCC.

## Materials and methods

### Patients

We retrospectively reviewed the medical records of patients with stage I OCCC who were treated at Nagoya University Hospital (Aichi, Japan) between 2005 and 2016. We identified ten patients who had experienced recurrence (cases 1–10) and ten age-matched patients who had never experienced recurrence (cases 11–20). Five patients underwent debulking surgery after recurrence, and we used their FFPE samples of cancer and normal ovary. Moreover, we used the recent fresh-frozen surgical samples of five patients with OCCC, including three patients with stage I cancer (cases 21–23) and two patients with stage III cancer (cases 24–25). All cases were histologically confirmed to be OCCC.

We established PDX mouse models using tissues from cases 21–23. Fresh surgical tissue was sectioned into ~3 mm^3^ pieces and implanted subcutaneously into a 5-week-old female NOD.Cg-*Prkdc*^*scid*^*Il2rg*^*tm1Wjl*^/SzJ mouse (Charles River Laboratories Japan, Kanagawa, Japan). The generation harboring the patient-derived material was termed F_1_, with subsequent generations numbered consecutively (e.g., F_2_–F_4_).

This study protocol was approved by the Ethics Committee of our institute (Approval Nos. 2015-0237, 2017-0053, and 2017-0497). We obtained written informed consent from all patients.

### RNA extraction and miRNA sequencing

Total RNA was extracted from eight 5-µm-thick sections of FFPE samples using an miRNeasy FFPE Kit (Qiagen, Hilden, Germany) and from fresh-frozen samples using an miRNeasy Mini Kit (Qiagen). The total RNA concentration was measured using a NanoDrop spectrophotometer (Thermo Fisher Scientific, Waltham, MA).

Comprehensive miRNA sequencing was performed according to the method described in our previous report [42]. Briefly, data analysis was performed using the CLC Genomics Workbench version 9.5.3 program (Qiagen), RStudio (RStudio, Boston, MA), and R software (ver. 3.5.0). To visualize a heatmap, miRNAs with low-normalized expression (<500 reads) were excluded. The raw data are shown in Supplementary Tables S2–S4.

### qPCR

For miRNAs, TaqMan Advanced miRNA cDNA Synthesis Kit, TaqMan Fast Advanced Master Mix, and TaqMan Advanced miRNA Assays (Assay IDs 478961_mir, 478964_mir, 478963_mir, and 479397_mir; Thermo Fisher Scientific) were used. For mRNA, a ReverTra Ace qPCR RT Kit (Toyobo, Osaka, Japan) and TB Green Premix Ex Taq (Takara Bio, Shiga, Japan) were used. Specific primers were synthesized by Hokkaido System Science (Hokkaido, Japan), and the primer sequences were described in Supplementary Fig. 2A. Then, qPCR was performed using Mx3000P (Agilent Technologies, Santa Clara, CA), and the detailed thermal profile was described in Supplementary Fig. 2B. Each experiment was performed triplicate and repeated at least three times.

### Cell lines

ES-2, TOV-21G, and SKOV3 cells were purchased from the ATCC (Manassas, VA), and A2780 and A2780cis cells were purchased from the ECACC (Porton Down, UK). These cell lines were maintained in RPMI (Nacalai Tesque, Kyoto, Japan) containing 10% fetal bovine serum (Sigma-Aldrich, St. Louis, MO), penicillin, and streptomycin (Meiji Seika Pharma, Tokyo, Japan). These cell lines were confirmed to be negative for mycoplasma contamination, and cells were used in 5–30 passages for experiments.

### Transfection and an inhibitor

Single-stranded miRNA mimics for miR-508-3p, miR-509-3p, miR-509-3-5p, and miR-514a-3p were synthesized, and NC #1 was purchased from Bioneer (Daejeon, Korea). For siRNA experiments, two Silencer Select Pre-designed siYAP1 (No. 1; s534572 and No. 2; s536629) and Silencer NC No. 1 siRNA were used (Thermo Fisher Scientific). Cells were transfected with 20 nM mimic or 2 nM siRNA using Lipofectamine RNAi Max (Thermo Fisher Scientific) for 24 h, and used for further analysis. For co-transfection, the total amount of miRNA mimics was 20 nM. In addition, cells were treated with 1 μM verteporfin, a YAP1 inhibitor (R&D Systems, Minneapolis, MN).

### Cisplatin sensitivity analyses

For MTS assay, cells were seeded into 96-well plate and simultaneously treated as described previously (*n* = 4 for each condition). Then, medium was replaced with cisplatin (Nichi-Iko Pharmaceutical, Toyama, Japan) containing medium, and cells were incubated for 48 h. Finally, 10 μl of 5 mg/ml CellTiter 96 Aqueous One Solution (Promega, Madison, WI) was added to each well and incubated with cells for 3 h, and the optical density (OD) was determined using a spectrophotometer at a wavelength of 490 nm. Cell viability was calculated as “(OD_cisplatin_ − OD_blank_)/(OD_control_ − OD_blank_) × 100”. The IC50 value was calculated using the following equation: IC50 (μM) = 10^[log(*A*/*B*)×(50−*C*)/(*D*−*C*)+log(*B*)]^, where *A* and *B* represent the highest and the lowest concentrations (µM) to cover an estimated IC50 value, respectively. *C* and *D* represent the cell viability at concentration *B* and *D*, respectively.

For apoptosis assay, cells were seeded into six-well plate and treater as described previously (*n* = 3). After cisplatin treatment (7.5 or 20 μM, 48 h), apoptosis assay was performed using a MEBCYTO Apoptosis Kit (Annexin V-FITC Kit; Medical & Biological Laboratories, Nagoya, Japan). A FACS Canto II flow cytometer (BD Biosciences, San Jose, CA) was used for detection. Early apoptotic cells are Annexin V-positive and PI-negative, whereas late apoptotic cells are Annexin V/PI-double-positive.

For clonogenic assay, 300 transfected cells were seeded into six-well plates (*n* = 6). Soon after the attachment of the cells to the dishes, the cells were treated with 1 μM cisplatin and incubated for 8 days. Then, cells were fixed and stained using a mixture of 4% paraformaldehyde and 0.5% crystal violet, and the colonies were counted.

Each experiment was performed at least three times.

### mRNA sequencing

ES-2 cells were transfected with the NC mimic or a combination of miR-509-3p and miR-509-3-5p in triplicate. Then, mRNA sequencing was performed by Riken Genesis (Tokyo, Japan). The obtained sequence data were trimmed, mapped to iGenomes Homo sapiens NCBI build 37.2, assembled, and normalized using fragment per kilobase of exon per million reads mapped (FPKM). Genes with low read coverage (<10 FPKM) were excluded, and significantly dysregulated genes were determined using an absolute log2 fold change exceeding 1 and an adjusted *p* value of < 0.05. The raw data are shown in Supplementary Table S5.

### Online tools

The location of miRNA precursors was referred from RNAcentral (https://rnacentral.org/). Putative target genes were referred from TargetScanHuman7.2 (http://www.targetscan.org/vert_72/), DIANA-microT-CDS (http://diana.imis.athena-innovation.gr/DianaTools/index.php?r=microT_CDS/index), miRWalk3.0 (http://mirwalk.umm.uni-heidelberg.de/), and miRanda (http://www.microrna.org/microrna/home.do). Moreover, DAVID (https://david.ncifcrf.gov/tools.jsp) was used for functional annotation.

### Western blotting

Protein was extracted using RIPA Lysis buffer (Millipore, Temecula, CA) with protease inhibitor cocktail tables (Roche Diagnostics, Indianapolis, IN) or NE-PER Nuclear and Cytoplasmic Extraction Reagents (Thermo Fisher Scientific). Protein was separated by 10% SDS-PAGE and transferred onto PVDF membranes. The membranes were blocked in 3% BSA/TBS-T, and the following antibodies were used: PhosphoPlus YAP (Ser127) Antibody Duet #46538 and Anti-rabbit IgG, HRP-linked Antibody #7074 (Cell Signaling Technology, Danvers, MA). Then, protein bands were examined using ELC Western Blotting Detection Reagents and the ImageQuant LAS 4000 mini (GE Healthcare, Backinghamshire, UK), and band intensities were quantified using ImageJ [43].

### Generation of a stable cell line overexpressing YAP1

ES-2 cells that constitutively expressed YAP1 were established by retrovirus infection. pQCXIP vectors (Takara Bio) that encoded its complementary DNA were transfected into 293T cells in combination with the pVPack-GP and pVPack-Ampho vectors (Stratagene, Tokyo, Japan) using calcium phosphate. Forty-eight hours after transfection, the supernatants were added to ES-2 cells along with 2 μg/ml polybrene (Sigma-Aldrich), and infected cells were selected by incubating with 1 μg/ml puromycin for 2 days.

### Immunohistochemistry

Immunohistochemistry was performed according to the method described in our previous report [44]. Briefly, an anti-YAP1 antibody (HPA070359; Sigma-Aldrich), a Histofine SAB-PO(R) kit, and 3, 3′-diaminobenzidine substrate-chromogen (Nichirei, Tokyo, Japan) were used.

### Statistical analysis

Statistical analysis was performed with SPSS version 26 (SPSS Inc., Chicago, IL). Student’s *t* test was used to determine the significance of differences between the means of two sets of data. The Kruskal–Wallis test was used to determine the significance of differences between three or more independent samples. A *p* value of < 0.05 was considered statistically significant.

## Supplementary information

Supplementary legends

Supplementary Fig. S1

Supplementary Fig. S2

Supplementary Table S1

Supplementary Table S2

Supplementary Table S3

Supplementary Table S4

Supplementary Table S5
