# Supplementary material for: Expression of the chrXq27.3 miRNA cluster in recurrent ovarian clear cell carcinoma and its impact on cisplatin resistance
Source: Oncogene. 2021 Jan 8;40(7):1255–68. doi: 10.1038/s41388-020-01595-3 (PMC7892337; doi:10.1038/s41388-020-01595-3)
Supplement: Supplementary file 1 — Supplementary legends [file 41388_2020_1595_MOESM1_ESM.docx]

**Supplementary legends**

**Supplementary Fig. S1. The impact of co-transfection of miR-509-3p and miR-509-3-5p in A2780cis cell line.**

**(A)** Expression of miR-509-3p and miR-509-3-5p in transfected A2780cis cells measured using qPCR. **(B)** Cisplatin sensitivity of A2780cis cells measured using MTS assay. The viability of the two miRNAs transfected cells was compared with that of NC transfected cells using Student’s *t*-test. **(C)** Apoptosis assay of transfected A2780cis cells treated with 20 µM cisplatin. The percentage of apoptotic cells was compared using Student’s *t*-test. **(D)** Validation of YAP1 suppression following co-transfection of miR-509-3p and miR-509-3-5p at the transcriptional and protein levels. Relative expression of *YAP1/GAPDH* was compared using Student’s *t*-test. **(E)** Cisplatin sensitivity of siYAP1-transfected A2780cis cells measured using MTS assay. The viability of treated cells was compared with that of cells transfected with siCtrl using Student’s *t*-test. Error bars represent standard errors of the mean. **p* < 0.05 and ***p* < 0.01.

**Supplementary Fig. S2. Primer sequences and protocols for qPCR.**

**(A)** Primer sequences **(B)** Protocols for qPCR

**Supplementary Table S1. List of significantly dysregulated genes targeted by miR-509-3p or miR-509-3-5p.**

**Supplementary Table S2. miRNA profiles of FFPE samples (cases 1–20).**

**Supplementary Table S3. miRNA profiles of fresh-frozen samples (21–25).**

**Supplementary Table S4. miRNA profiles of ovarian cancer cell lines.**

**Supplementary Table S5. Transcriptome profiles of transfected ES-2 cells.**
