# Supplementary figures and images for: Expression of the chrXq27.3 miRNA cluster in recurrent ovarian clear cell carcinoma and its impact on cisplatin resistance
Source: Oncogene. 2021 Jan 8;40(7):1255–68. doi: 10.1038/s41388-020-01595-3 (PMC7892337; doi:10.1038/s41388-020-01595-3)

# Supplementary Fig. S1

**A**

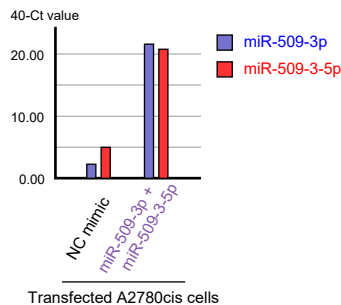

**B**

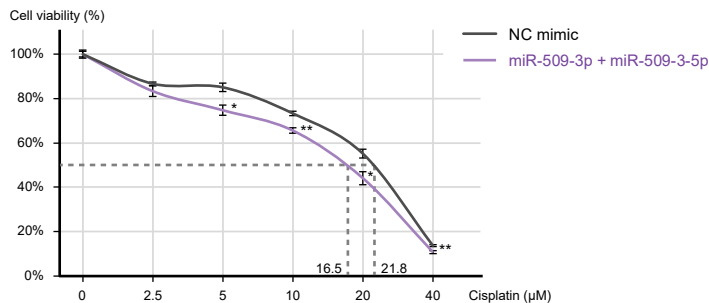

**C**

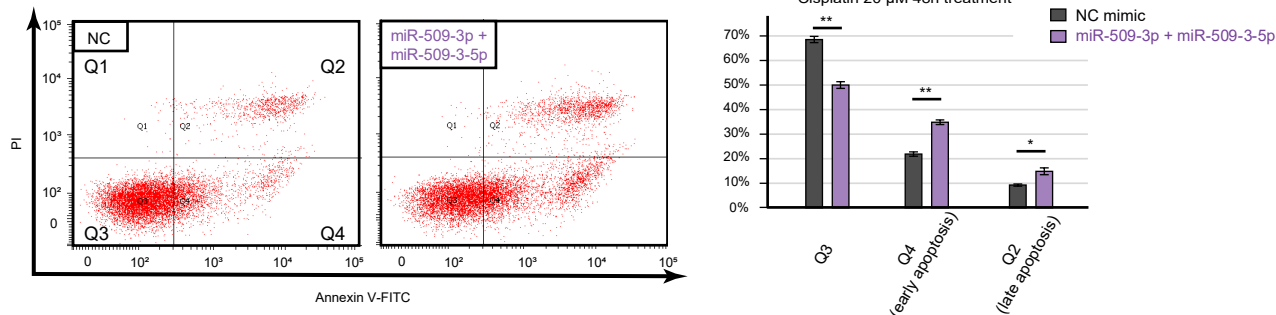

**D**

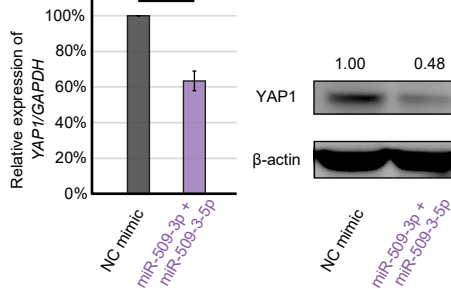

**E**

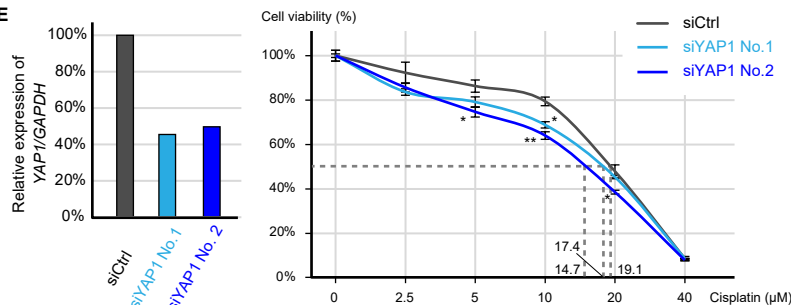

Supplement: Supplementary file 2 — Supplementary Fig. S1 [file 41388_2020_1595_MOESM2_ESM.pdf]
