# Supplementary material for: Expression of the chrXq27.3 miRNA cluster in recurrent ovarian clear cell carcinoma and its impact on cisplatin resistance
Source: Oncogene. 2021 Jan 8;40(7):1255–68. doi: 10.1038/s41388-020-01595-3 (PMC7892337; doi:10.1038/s41388-020-01595-3)
Supplement: Supplementary file 3 — Supplementary Fig. S2 [file 41388_2020_1595_MOESM3_ESM.pdf]

Supplementary Fig. S2

A

Name	Sequence
Forward primer for YAP1	AGAACAATGACGACCAATAGCTC
Reverse primer for YAP1	GCTGCTCATGCTTAGTCCAC
Forward primer for GAPDH	AAATGGGGTGAGGCCGGT
Reverse primer for GAPDH	ATTGCTGACAATCTTGAGTGA

B

For miRNA

Step	Temp.	Time	Cycles
Enzyme activation	95°C	10 min	1
Denature	95°C	15 s	40
Anneal/Extend	60°C	60 s	

For mRNA

Step	Temp.	Time	Cycles
Enzyme activation	95°C	30 s	1
Denature	95°C	5 s	40
Anneal/Extend	60°C	30 s	
